# Adequacy of Cancer-Related Pain Treatments and Factors Affecting Proper Management in Ayder Comprehensive Specialized Hospital, Mekelle, Ethiopia

**DOI:** 10.1155/2020/2903542

**Published:** 2020-09-24

**Authors:** Kald Beshir Tuem, Leake Gebremeskel, Kibrom Hiluf, Kbrom Arko, Haftom Gebregergs Hailu

**Affiliations:** ^1^Department of Pharmacology and Toxicology, School of Pharmacy, College of Health Sciences, Mekelle University, Mekelle, Ethiopia; ^2^Department of Pharmacy, College of Health Sciences, Aksum University, Aksum, Ethiopia; ^3^Department of Oncology, School of Medicine, College of Health Sciences, Mekelle University, Mekelle, Ethiopia

## Abstract

**Background:**

Cancer-related pain (CRP) is a major problem with a potential negative impact on quality of life of the patients and their caregivers.

**Purpose:**

To assess the adequacy of cancer-related pain management in Ayder Comprehensive Specialized Hospital (ACSH). *Methodology*. A facility-based cross-sectional study design was conducted in ACSH from January to March 2019. A well-structured professional-assisted questionnaire using Brief Pain Inventory-Short Form (BPI-SF) was used to collect data concerning the severity of pain, functioning interference, and adequacy of pain management in cancer patients. Data were analyzed using SPSS v.21.

**Result:**

Out of 91 participants, 47 (51.6%) were male and 52 (57.1%) were between the age group of 18–45, with the mean age of 44.8 ± 13.6 years. According to the pain assessment tool (BPI), 85 (93.4%) patients experienced pain and 90 (98.9%) patients had activity interference; negative pain management index (PMI) was observed in 40 (43.95%) patients, showing that 43.95% were receiving inadequate pain management. Out of 38 patients who received no analgesics, 35.2% were found to have inadequate pain management, whereas those who took strong opioids had 100% effective pain management and the majority of the patients were in stage III. Among 38 (41.76%) only 20 (52.63%) received adequate pain management, based on patients' self-report in which 18.7% of the participants stated that they got 30% pain relief and only 1.1% got 90% relief. The predictors of undertreatment were presence of severe pain, metastasis, comorbidity, and stage of the cancer and could also be due to the educational level and monthly income, as evidenced by significant association.

**Conclusion:**

This study suggests that cancer pain management in ACSH was sufficient for only 56%. However, large numbers of individuals are suffering from a manageable pain. Hence, remedial action should be taken, including increasing awareness of symptom management in medical staff and incorporating existing knowledge into routine clinical practice.

## 1. Introduction

Cancer is a disease condition in which the body's cells begin to grow and proliferate in an uncontrollable way [1, 2]. The major cause of cancer, 90–95% of cases, is due to genetic mutations from environmental factors and the remaining 5–10% is by inherited genetics [3]. The pathophysiology of cancer is very complex in which malignancy occurs through overexpression of normal oncogenes or underexpression of tumor suppressor genes. The report from the World Health Organization (WHO) [4] shows that cancer is the major cause of morbidity and mortality globally, 18.1 million new cases and 9.6 million deaths annually. Popat et al. [5] highlighted that cancer and other noncommunicable diseases are estimated to surpass some infectious diseases as leading causes of death in the African region by the year 2030. In Ethiopia, it accounts for 4% of all deaths and approximately 60,000–125,000 cancer patients visit the Tikur Anbessa Specialized Hospital (TASH) oncology unit annually [2].

According to previous studies, greater than half of the patients with cancer experience pain from moderate to severe intensity [6, 7]. Cancer-related pain (CRP) can be nociceptive pain which comes from the actual damage of nonneural tissues and neuropathic pain which is caused by damage of the somatosensory nervous system [3, 6, 8]. Pain assessment is an integral component of cancer pain management which aims at identifying pain characteristics, pain etiology, specific pain syndromes, and analgesic targets using pain management index (PMI) [9]. To manage CRP effectively, the WHO has developed a 3-step pain ladder which includes the use of a nonopioid (paracetamol) for mild pain, a weak opioid (Codeine) for moderate pain, and a strong opioid (morphine) for severe pain [10].

Despite the availability of many guidelines for the treatment of CRP, patients usually receive inadequate pain management as highlighted by studies from different parts of the world. A report in Japan shows physicians undertreated CRP in 70% of patients [11]. In a cross-sectional study in Portugal, patients' pain management index status suggests that cancer-related pain treatment was insufficient in 25.6% of patients and about 1 in 4 patients was poorly treated at first consultation in the CRP clinic [12]. Moreover, a meta-analysis showed that pooled prevalence rates of cancer-related pain in patients treated with disease-modifying treatment and in advanced terminal disease were found to be 55% and 64%, respectively [13, 14]. As studies indicate, the burden of cancer-related pain in developing countries is too high where approximately 80% of individuals die from cancer-related moderate or severe pain lasting for 90 days [4, 15]. Although adequacy of CRP management study is too limited in Ethiopia, one study conducted in Gondar showed that 65% of patients did not get adequate CRP management [16]. As the adequacy of CRP treatment is not well studied in Ayder Comprehensive Specialized Hospital (ACSH) so far, we aimed to evaluate the adequacy of CRP management and its determinants. Hence, this research could be utilized as a starting point for further research and improvement of CRP management in the hospital oncology center.

## 2. Methods

### 2.1. Study Area

This research was done at Mekelle University, Ayder Comprehensive and Specialized Hospital, located in Tigray, Northern Ethiopia. The hospital was established to provide educational and medical services in 2008 to around 9 million people from Tigray and neighboring regions such as Afar and Northeastern Amhara and Eritrea. It renders a variety of medical services for all age groups in both inpatient and outpatient departments. Ayder Comprehensive and Specialized Hospital is the second largest hospital in the country. It has about 500 inpatient beds in all departments including the oncology unit [17].

### 2.2. Study Design and Period

A cross-sectional study design was conducted from January 01 to March 30, 2019, to assess CRP in the oncology unit of ACSH.

### 2.3. Study Population

Study population included all adult cancer patients visiting Ayder Comprehensive Specialized Hospital during the study period.

### 2.4. Inclusion Criteria

Cancer patients who meet the following criteria were included in the study: eighteen years old and greater; patients diagnosed with any kind of cancer; and all patients in the outpatient and inpatient wards of the oncology unit at specified time duration.

### 2.5. Exclusion Criteria

Terminally ill patients, patients with neurologic disorders, and individuals who refuse to participate in the study were excluded.

### 2.6. Sample Size Determination

All patients who attended the outpatient and inpatient oncology department at the specified study period were included in the study in a census manner as long as they meet the inclusion criteria.

### 2.7. Data Collection Procedure

Data were collected using the Brief Pain Inventory-Short Form (BPI-SF) [18] and chart review. The questionnaire-based data collection has an 8-item questionnaire which was applied to assess the impact and severity of pain on the daily functioning of the patient. The eight items of the questionnaire, BPI-SF, are described as follows: item number 1 is used to indicate a specific part of the body or coverage of pain where patients feel; items numbers 2 to 5 assess the severity of pain. The calculation of pain severity score was performed by dividing the total score from item numbers 2 to 5 by 4 [19], which gives severity out of ten. The type of medication given and the percentage of pain relief of patients were described by item numbers 6 and 7. Pain interference in seven daily activities was measured using item number 8 (8.1 up to 8.7), and pain interference was calculated by dividing the sum of the scores for each query (from 8.1 up to 8.7) by 7. This also produces an interference score out of ten in which items of interference were put with 0–10 scales denoting that 0 shows no interference, whereas 10 indicates full interference. Finally, using BPI-SF, severity and interference pain was classified into 4 groups: no pain (0), mild (1 up to 3), moderate (4 up to 7), and severe (8 up to 10).

Much information was collected from the patient including sociodemographic variables, comorbidity status, patient diagnoses, cancer location and stages, presence or absence of metastases, treatment modality, and number of drugs given and analgesics prescribed. The questionnaire was first translated into Tigrigna (the locally official language) and then translated back to English to verify accuracy. Clinical data were gathered from chart review, and type and severity of pain grading, analgesic use with percentage of pain interference, and relief of pain were collected by interviewing patients using the BPI-SF data collection tool.

According to the type of antipain medication(s) patient uses, scores were given as follows: 0 (no analgesic drug), 1 (nonopioid antipain drug), 2 (weak opioid), and 3 (strong opioid), and then PMI was determined. Four levels of analgesic medications were estimated by the potency: (0) no order for antipain drug, (1) nonopioid (nonsteroidal anti-inflammatory drugs), (2) weak opioid (codeine), and (3) strong opioid (morphine), and then potency of drugs was compared with “ worst pain.” No pain was scored as “0,” mild pain “1,” moderate pain “2,” and severe pain “3.” Finally, the PMI is determined by subtracting the pain level from the analgesic level in which the values go from −3 (severe pain with no analgesic medication) to +3 (morphine use and no pain reported). Therefore, inadequate pain management is considered when negative PMI is scored.

## 3. Variables

### 3.1. Dependent Variable

  Cancer-related pain management
  Pain-related interferences

### 3.2. Independent Variables

  Age, sex, religion, ethnicity, educational status, family size, residence, income, marital status, stage of cancer, type of cancer, and site of cancer-related pain.
  Antipain medication administration

### 3.3. Data Management and Analysis

Statistical analysis was done using SPSS version 21. Descriptive statistics was used to summarize demographic characteristics, pain type, and number and type of analgesics given to the patient. Adequacy of cancer pain management and pain interference were cross tabulated to compare the magnitude among different variables. Association was tested between independent and dependent variables using the chi-square test. Significance level was set by fixing*p* < 0.05.

### 3.4. Data Quality Control

The questionnaire was pretested in a total of 20 individuals at a hospital which is not a study site (Mekelle hospital). The completeness of the questionnaire was checked step by step by the data collectors and supervisors and further counterchecked by the principal investigator daily.

## 4. Result

A total of ninety-one (91) participants were included in the study. Nearly half of the patients (47 (51.6%)) were male while the rest were females (48.4%). Majority of them (74 (81.3%)) were Orthodox Christians. The mean age was 44.8 ± 13.6 years ranging from 19 to 72 years, sixty-three (69.2%) were from urban areas. The mean income of a family was 3590.2 ± 2336.5, and the family size of the participants was 4.97 ± 3.06 (Table 1).

Of the total number of participants, 81 (89%) were admitted. Only 6 (6.6%) patients had comorbidities. As the study indicates, 22 (22%), 38 (41.8%), and 33 (36.3%) patients were in stages II, III, and IV, respectively. It was observed that 42 (46.1%) of the patients were with metastasized cancer. Nearly fifty percent of the patients (50 (54.9%)) experienced a moderate severity of pain. Most of the patients (59 (64.8%)) received chemotherapy plus surgical intervention (Table 2).

With regard to the type of pain, most patients experienced a mixed type of pain (44 (48.4%)) followed by nociceptive pain (16 (17.6%)) (Table3).

The commonest site of CRP was in the intestinal area 31 (34.1%), followed by genitourinary (18 (19.8%)), and least at the amputated site (1 (1.1%)) (Table 4).

According to the information collected using the pain assessment tool (BPI), 85 (93.4%) patients experienced pain, of which 50 (54.9%) had moderate pain, while only 6 (6.6%) felt no pain. Likewise, almost all (90 (98.9%)) patients had pain functioning interference; among those, 36 (39.6%) patients faced moderate pain and 32 (35.2%) felt severe pain (Table 5).

**Figure 1:**
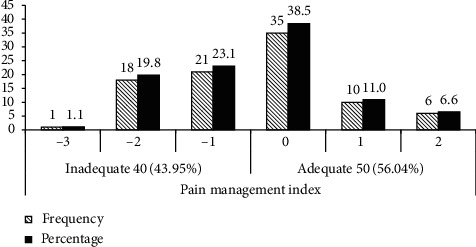
PMI and the number of patients with adequate or inadequate management of pain at the oncology unit of ACSH, Mekelle, Ethiopia.

While investigating the adequacy of pain treatment, negative PMI was observed in 40 (43.95%) patients showing that they were receiving inadequate pain management. Most of the patients (51 (56.04%)) had received adequate management of pain (Figure 1).

**Figure 2:**
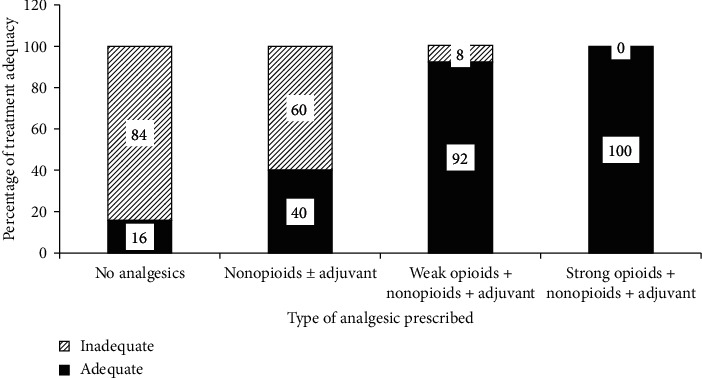
Percentage of CRP treatment adequacy within types of analgesics prescribed at the oncology unit of ACSH, Mekelle, Ethiopia.

Out of the 38 patients who received no analgesics, 32 (84%) had inadequate pain management while 100% effective pain management in patients taking strong opioids (Figure 2).

Upon crosstab and Pearson chi-square analysis of the adequacy of CRP treatment and pain functioning interference, it was revealed that pain was more significantly adequately managed in patients with the following characteristics: age ranging from 18 to 45 years, males, orthodox, married, illiterate, urban dwellers, having >5000 monthly income, stage III and stage IV of the disease, absence of metastasis, being treated with chemotherapy plus surgery, absence of comorbidity, having moderate pain severity, and being on strong opioids + nonopioids + adjuvant. With regard to pain interference presence on functioning, moderate to severe interference of pain was most likely to be present in patients with the following characteristics: age ranging from 18 to 45 years, males, orthodox, divorced, illiterate, urban dwellers, having ≤ 2000 monthly income, stage III of the disease, presence of metastasis, being treated by combination therapy, absence of comorbidity, presence of history of pain, moderate pain severity, and being on strong opioids + nonopioids + adjuvant (Tables 6–8).

**Figure 3:**
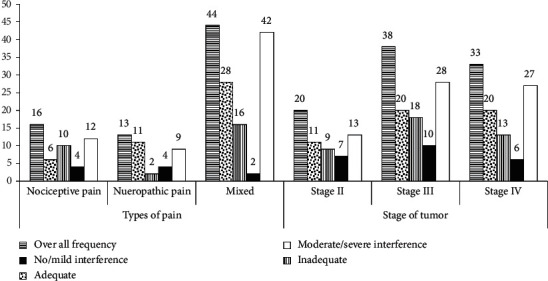
Frequency of type of pain and stage of tumor in relation with treatment adequacy and pain interference at the oncology unit of ACSH, Mekelle, Ethiopia (*n* = 91).

Forty-four (48.35%) patients had mixed type of pain, of which 28 (63.6%) were adequately treated and 42 (95.5%) had moderate to severe functioning interference. Regarding the staging of cancer, the majority of patients were on stage III 38 (41.76%), of which 20 (52.63%) received adequate pain management and 28 (73.7%) faced moderate to severe functioning interference (Figure 3).

**Figure 4:**
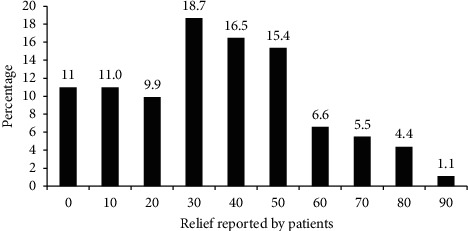
Percentage of relief reported by the patients at the oncology unit of ACSH, Mekelle, Ethiopia.

The patients were allowed to self-report their relief to the provided analgesics and only 18.7% of the patients have responded that the drugs prescribed reduced their pains by 30%. Relatively small percentage (1.1%) of the patients got 90% relief while 11% of the participants exercised 0% effective pain relief (Figure 4).

## 5. Discussion

Pain is one of the most frequent and distressing symptoms experienced by cancer patients, and it affects their quality of life [11]. However, evidence from clinical practice indicates that pain of cancer patients may be treated in up to 90% cases with the current analgesics [20–22]. Limited researches have been done on the adequacy of CRP in Ethiopia, and to the best of our knowledge, there is no study conducted in ACSH. It is believed that this study will be used as a baseline for further research regionally and nationally.

In this study, a total of 91 cancer patients participated. It was found that 93.4% of them had cancer-related pain. This is comparable with the study result from Gondar, Ethiopia, which reported 91.6% of cancer patients experience CRP [16], but much higher as compared to the study result from Portugal, which claimed only 25% of patients had CRP [12]. Previous studies have indicated that this difference could be due to low awareness of clinicians on assessment of CRP, lack of updated guidelines, and shortage of analgesics such as morphine [23, 24].

According to this study, 43.9% of patients received inadequate pain management, which is lower in comparison to a study result from Gondar which reported 65% of cancer patients had received inadequate pain management [16]. However, it was found that our result was higher as compared to a research performed in Portugal (25.6%) [12] and in Ghana (26.4%) [25]. The main determinants in CRP treatment are patient-related factors [26, 27], disease conditions including stage of cancer (all patients were with stage II and above) and presence of metastasis (42%), and healthcare provider-related factors [28] that may contribute to the undertreatment of cancer-related pain, which were not assessed in this study.

The study revealed that a significant association of inadequacy of pain management was observed with marital status, level of education, presence of comorbidity, types of pain and pain severity, and activity functioning interference. This finding was similar to the study conducted in the University of Gondar [16].

Cancer-related pain may have interference with daily activities. This study found that almost all (98.9%) cancer patients experienced cancer pain interference in their daily activities. This figure is higher when compared to the result of a study from Northern Ethiopia in which it was found to be 89.2% [16].

Interference of CRP with daily activities is highly affected by stage of cancer. This study found that cancer patients with stages II, III, and IV have no/mild and moderate/severe pain interferes with their daily activities (no/mild, moderate/severe pain: 7.7%, 14.3%; 11%, 30.8%; and 6.6%, 29.4%, respectively). This study's result was comparable with findings of the study from Gondar (no/mild, moderate/severe pain: 7.2%, 16.9%; (18.1%, 26.5%; and 7.2%, 15.7%, respectively). However, the severe interference of pain in stage IV was higher in our study (29.4%) relative to that of Gondar (15.7%) [16].

## 6. Conclusion

In conclusion, cancer-related pain management in Ayder Comprehensive Specialized Hospital is inadequate, and some of the patient's pain was not managed appropriately as indicated by negative pain index. Assessment of the knowledge and perception of health professionals working in the oncology unit of ACSH and the availability and affordability of antipain medications should be done to find out their role in the inadequacy of CRP management. In addition, the hospital should develop guidelines and drug use policies specifically for CRP management, and in-service training regarding CRP management should be given to health care providers who are working in cancer centers.

## Figures and Tables

**Table 1:** Demographic details of cancer patients at the oncology unit of ACSH, Mekelle, Ethiopia (*n* = 91).

Variables	Frequency	Percentage
*Gender*		
Male	47	51.6
Female	44	48.4

*Age category in years∗*		
18–45	52	57.1
46–65	33	36.3
≥65	6	6.6

*Residence*		
Rural	28	30.8
Urban	63	69.2

*Region*		
Tigray	89	97.8
Afar	1	1.1
Others	1	1.1

*Religion*		
Orthodox	74	81.3
Protestant	9	9.9
Muslim	8	8.8

*Occupation*		
Student	11	12.1
Governmental	12	13.2
Merchant	15	16.5
Farmer	33	36.3
Private	9	9.9
None	11	12.1

*Marital status*		
Married	65	71.4
Single	14	15.4
Widowed	6	6.6
Divorced	6	6.6

*Income range∗∗*		
≤2000	18	19.8
2001–5000	10	11
>5000	14	15.4
Missing (no data)	49	

*Family size∗∗∗*		
1–3	13	14.3
4–6	33	36.3
>6	31	34.1
0	14	15.4

Mean = *∗*44.8 ± 13.6; *∗∗*3590.2 ± 2336.5; *∗∗∗*4.97 ± 3.06.

**Table 2:** Health-related variables of cancer patients at the oncology unit of ACSH, Mekelle, Ethiopia (*n* = 91).

Variables	Frequency	Percentage
*Stage*		
II	20	22
III	38	41.8
IV	33	36.3

*Type of patient*		
Admitted	81	89
Ambulatory	10	11

*Metastasis*		
Present	42	46.1
Absent	49	53.8

*Pain severity*		
No pain	6	6.6
Mild	27	29.7
Moderate	50	54.9
Severe	8	8.8

*Treatment modality*		
Chemo therapy only	32	35.2
Chemo therapy + surgery	59	64.8

**Table 3:** Type of pain experienced by cancer patients at the oncology unit of ACSH, Mekelle, Ethiopia (*n* = 91).

Type of pain	Frequency	Percentage
Nociceptive	16	17.6
Neuropathic	13	14.3
Mixed	44	48.4
Missing (no data)	12	13.2

**Table 4:** Common site of cancer-related pain among cancer patients at the oncology unit of ACSH, Mekelle, Ethiopia (*n* = 91).

Site of pain	Frequency	Percentage
Lung	14	15.4
Genitourinary	18	19.8
Head and neck	12	13.2
Intestine	31	34.1
Amputated site	1	1.1
Breast	4	4.4
Nonspecified	11	12.1

**Table 5:** Pain severity and pain interference among cancer patients at the oncology unit of ACSH, Mekelle, Ethiopia (*n* = 91).

Pain related variables		Frequency	Percentage
Severity of pain	No pain	6	6.6
	Mild	27	29.7
	Moderate	50	54.9
	Severe	8	8.8

Pain interference severity	No interference	1	1.1
	Mild	22	24.2
	Moderate	36	39.6
	Severe	32	35.2

Pain management index	2	6	6.6
	1	10	11.0
	0	35	38.5
	−1	21	23.1
	−2	18	19.8
	−3	1	1.1

Adequacy of pain management	Adequate	51	56.04
	Inadequate	40	43.95

**Table 6:** Relationship among sociodemographic variables and adequacy of management and pain interference at the oncology unit of ACSH, Mekelle, Ethiopia (*n* = 91).

	Adequacy of treatment, *n* (%)		Association	Pain functioning interference, *n* (%)			Association
	Adequate	Inadequate	*X* ^2^, *p* value	No/mild	Moderate/severe	*X* ^2^, *p* value
Age range				5.21, 0.074			1.44, 0.488
	18–45	33 (36.3)	19 (20.9)		15 (16.5)	37 (40.7)	
	46–65	17 (18.7)	16 (17.6)		6 (6.6)	27 (29.7)	
	>65	1 (1.1)	5 (5.5)		2 (2.2)	4 (4.4)	

Sex				2.39, 0.122			0.00, 0.953
	Male	30 (33.0)	17 (18.7)		12 (13.2)	35 (38.5)	
	Female	21 (23.1)	23 (25.3)		11 (12.1)	33 (36.3)	

Religion				2.20, 0.333			1.08, 0.584
	Orthodox	39 (42.9)	35 (38.5)		20 (22.0)	54 (59.3)	
	Protestant	7 (7.7)	2 (2.2)		1 (1.1)	8 (8.8)	
	Muslim	5 (5.5)	3 (3.3)		2 (2.2)	6 (6.6)	

Family size				3.83, 0.281			1.18, 0.758
	0	8 (8.8)	6 (6.6)		2 (2.2)	12 (13.2)	
	1–3	10 (11.0)	3 (3.3)		4 (4.4)	9 (9.9)	
	4–6	19 (20.9)	14 (15.4)		9 (9.9)	24 (26.4)	
	>6	14 (15.4)	17 (18.7)		8 (8.8)	23 (25.3)	

Occupation				4.86, 0.433			4.02, 0.547
	Student	8 (8.8)	3 (3.3)		1 (1.1)	10 (11.0)	
	Government employee	7 (7.7)	5 (5.5)		4 (4.4)	8 (8.8)	
	Merchant	10 (11.0)	5 (5.5)		6 (6.6)	9 (9.9)	
	Farmer	16 (17.6)	17 (18.7)		8 (8.8)	25 (27.5)	
	Private work	6 (6.6)	3 (3.3)		2 (2.2)	7 (7.7)	
	Not working	4 (4.4)	7 (7.7)		2 (2.2)	9 (9.9)	

Marital status				8.50, 0.037*∗*			6.40, 0.094
	Married	36 (39.6)	29 (31.9)		1 (1.1)	(101.0)	
	Single	8 (8.8)	6 (6.6)		4 (4.4)	8 (8.8)	
	Widowed	1 (1.1)	5 (5.5)		6 (6.6)	9 (9.9)	
	Divorced	6 (6.6)	0 (0)		8 (8.8)	25 (27.5)	

Education level				10.66, 0.014*∗*			0.50, 0.919
	Illiterate	18 (19.8)	25 (27.5)		12 (13.2)	31 (34.1)	
	Primary	13 (14.3)	2 (2.2)		3 (3.3)	12 (13.2)	
	Secondary school	10 (11.0)	4 (4.4)		3 (3.3)	11 (12.1)	
	College or university	10 (11.0)	9 (9.9)		5 (5.5)	14 (15.4)	

Residence				0.10, 0.751			0.32, 0.574
	Rural	15 (16.5)	13 (14.3)		6 (6.6)	22 (24.2)	
	Urban	36 (39.6)	27 (29.7)		17 (18.7)	46 (50.5)	

Income range				1.75, 0.417			0.22, 0.895
	≤2000	9 (9.9)	9 (9.9)		7 (7.7)	11 (12.1)	
	2001–5000	5 (5.5)	5 (5.5)		3 (3.3)	7 (7.7)	

**Table 7:** Relationship among clinical variables and adequacy of management and pain interference at the oncology unit of ACSH, Mekelle, Ethiopia (*n* = 91).

	Adequacy of treatment, *n* (%)		Association	Pain functioning interference, *n* (%)			Association
	Adequate	Inadequate	X^2^, *p* value	No/mild	Moderate/severe	X^2^, *p* value
Type of patient status				0.167, 0.683			7.173, 0.007*∗∗*
	Admitted	46 (50.5)	35 (38.5)		17 (18.7)	64 (70.3)	
	Ambulatory	5 (5.5)	5 (5.5)		6 (6.6)	4 (4.4)	

Site of cancer				11.581, 0.072			12.092, 0.600
	Genitourinary cancer	5 (5.5)	12 (13.2)		7 (7.7)	10 (11.0)	
	Gastrointestinal cancer	11 (12.1)	13 (14.3)		10 (11.0)	14 (15.4)	
	Breast cancer	3 (3.3)	1 (1.1)		0 (0)	4 (4.4)	
	Head and neck cancer	9 (9.9)	3 (3.3)		1 (1.1)	11 (12.1)	
	Bronchopulmonary cancer	8 (8.8)	5 (5.5.)		1 (1.1)	12 (13.2)	
	Follicular lymphoma	3 (3.3)	0 (0)		0 (0)	3 (3.3)	
	Others	12 (13.2)	6 (6.6)		4 (4.4)	14 (15.4)	
Stage of tumor				0.467, 0.792			1.902, 0.386
	Stage II	11 (12.1)	9 (9.9)		7 (7.7)	13 (14.3)	
	Stage III	20 (22.0)	18 (19.8)		10 (11.0)	28 (70.8)	
	Stage IV	20 (22.0)	13 (14.3)		6 (6.6)	27 (29.7)	

Metastasis				1.566, 0.457			7.459, 0.024*∗*
	Present	22 (24.2)	19 (20.9)		5 (5.5)	36 (39.6)	
	Absent	29 (31.9)	20 (22.0)		18 (19.8)	31 (34.1)	

History of treatment modality				1.839, 0.175			1.113, 0.292
	Chemotherapy	21 (23.1)	11 (12.1)		6 (6.6)	26 (28.6)	
	Chemotherapy + surgery	30 (33.0)	29 (31.9)		17 (18.7)	42 (46.2)	

Comorbidity				5.038, 0.025*∗*			0.252, 0.616
	Present	6 (6.6)	0 (0)		1 (1.1)	5 (5.5)	
	Absent	45 (49.5)	40 (44.0)		22 (24.2)	63 (69.2)	

*∗∗*Significant at *p* < 0.01; *∗*significant at *p* < 0.05.

**Table 8:** Relationship among pain-related parameters and adequacy of management and pain interference at the ACSH oncology unit, Mekelle, Ethiopia, April 2019 (*n* = 91).

	Adequacy of treatment, *n* (%)		Association	Pain functioning interference *n* (%)			Association
	Adequate	Inadequate	*X* ^2^, *p* value	No/mild	Moderate/severe		*X* ^2^, *p* value
History of pain				5.716, 0.017			19.395, 0.000*∗∗∗*
	Present	44 (48.4)	26 (28.6)		10 (11.0)	60 (65.9)	
	Absent	7 (7.7)	14 (15.4)		13 (14.2)	8 (8.8)	

Types of pain				11.340, 0.010*∗∗*			31.225, 0.000*∗∗∗*
	Nociceptive pain	6 (6.6)	10 (11.0)		4 (4.4)	12 (13.2)	
	Neuropathic pain	11 (12.1)	2 (2.2)		4 (4.4)	9 (9.9)	
	Mixed	28 (30.8)	16 (17.6)		2 (2.2)	42 (46.2)	

Pain severity				5.508, 0.138			45.536, 0.000*∗∗∗*
	No pain	6 (6.6)	0 (0)		6 (6.6)	0 (0)	
	Mild	13 (14.3)	14 (15.4)		15 (16.5)	12 (13.2)	
	Moderate	28 (30.8)	22 (24.2)		2 (2.2)	48 (52.7)	
	Severe	4 (4.4)	4 (4.4)		0 (0)	8 (8.8)	

Class of analgesics administered				53.253, 0.000*∗∗*			17.479, 0.001*∗∗*
	No analgesics	6 (6.6)	32 (35.2)		17 (18.7)	21 (23.1)	
	No opioids ± adjuvant	4 (4.4)	6 (6.6)		0 (0)	10 (11.0)	
	Weak opioids ± nonopioids ± adjuvant	24 (26.4)	2 (2.2)		1 (1.1)	25 (27.5)	
	Strong opioids ± nonopioids ± adjuvant	17 (18.7)	0 (0)		5 (5.5)	12 (13.2)	

Pain interference severity				8.799. 0.032*∗*			91.0, 0.000*∗∗∗*
	No interference	1 (1.1)	0 (0)		1 (1.1)	0 (0)	
	Mild	11 (12.1)	11 (12.1)		22 (24.2)	0 (0)	
	Moderate	15 (16.5)	21 (23.8)		0 (0)	36 (39.6)	
	Severe	24 (26.4)	8 (8.8)		0 (0)	32 (35.2)	

*∗∗∗*Significant at *p* < 0.001; *∗∗*significant at *p* < 0.01; *∗*significant at*p* < 0.05.

## Data Availability

The data used to support the findings of this study are available from the corresponding author upon request.

## Abbreviations

BPI-SF: Brief Pain Inventory-Short Form
CRP: Cancer-related pain
CT: Computerized tomography
ECG: Electrocardiograph
ECHO: Echocardiography
EPIC: European pain in cancer
NCCN: National Comprehensive Cancer Network
PMI: Pain management index
WHO: World Health Organization.
